# Lag Time Remains with Newer Real-Time Continuous Glucose Monitoring Technology During Aerobic Exercise in Adults Living with Type 1 Diabetes

**DOI:** 10.1089/dia.2018.0364

**Published:** 2019-05-22

**Authors:** Dessi P. Zaharieva, Kamuran Turksoy, Sarah M. McGaugh, Rubin Pooni, Todd Vienneau, Trang Ly, Michael C. Riddell

**Affiliations:** ^1^ Kinesiology and Health Science, Faculty of Health, Muscle Health Research Centre, York University, Toronto, Canada.; ^2^ Department of Biomedical Engineering, Illinois Institute of Technology, Chicago, Illinois.; ^3^ Insulet Canada Corporation, Oakville, Canada.; ^4^ Insulet Corporation, Billerica, Massachusetts.; ^5^ LMC Diabetes and Endocrinology, Toronto, Canada.

**Keywords:** Continuous glucose monitoring, Accuracy, Self-monitoring of blood glucose, Type 1 diabetes, Sensors, Time lag

## Abstract

***Background:*** Real-time continuous glucose monitoring (CGM) devices help detect glycemic excursions associated with exercise, meals, and insulin dosing in patients with type 1 diabetes (T1D). However, the delay between interstitial and blood glucose may result in CGM underestimating the true change in glycemia during activity. The purpose of this study was to examine CGM discrepancies during exercise and the meal postexercise versus self-monitoring of blood glucose (SMBG).

***Methods:*** Seventeen adults with T1D using insulin pump therapy and CGM completed 60 min of aerobic exercise on three occasions. A standardized meal was given 30 min postexercise. SMBG was measured during exercise and in recovery using OmniPod^®^ Personal Diabetes Manager (PDM; Insulet, Billerica, MA) with built-in glucose meter (FreeStyle; Abbott Laboratories, Abbott Park, IL), while CGM was measured with Dexcom G4^®^ with 505 algorithm (*n* = 4) or G5^®^ (*n* = 13), which were calibrated with subjects' own PDM.

***Results:*** SMBG showed a large drop in glycemia during exercise, while CGM showed a lag of 12 ± 11 (mean ± standard deviation) minutes and bias of −7 ± 19 mg/dL/min during activity. Mean absolute relative difference (MARD) for CGM versus SMBG was 13 (6–22)% [median (interquartile range)] during exercise and 8 (5–14)% during mealtime. Clarke error grids showed CGM values were in zones A and B 94%–99% of the time for SMBG.

***Conclusion:*** In summary, the drop in CGM lags behind the drop in blood glucose during prolonged aerobic exercise by 12 ± 11 min, and MARD increases to 13 (6–22)% during exercise as well. Therefore, if hypoglycemia is suspected during exercise, individuals should confirm glucose levels with a capillary glucose measurement.

## Introduction

Real-time continuous glucose monitoring (CGM) devices have been shown to detect greater glucose variability, rate of change (ROC) in glucose concentration, and incidence of hypoglycemia compared with self-monitoring of blood glucose (SMBG) using a traditional glucose meter and capillary blood sampling.^1,2^ Since CGM systems can provide insight into glucose patterns and overall diabetes management, these devices have recently been included in the clinical guidelines and standards of care for patients living with diabetes, particularly if they are dosing insulin.^3,4^

One common measure of CGM accuracy is mean absolute relative difference (MARD), which is the mean difference, expressed in absolute terms, between the reference (often SMBG) and interstitial glucose divided by the reference. Successive generations of devices have improved sensor accuracy when comparing with laboratory standards (e.g., Yellow Springs Instruments [YSI] glucose analyzer), thereby leading to nonadjunctive status, so that insulin dosing and decision-making around hypoglycemia treatment can be made using some of these devices.^3,5,6^ With newer CGM technology, whether MARD remains stable throughout dynamic changes in glucose (often associated with exercise and meal ingestion) is up for debate.^7^

In an earlier field study,^8^ CGM was shown to help guide patients on when to initiate carbohydrate feeding during physical activity in active adolescents and young adults with type 1 diabetes (T1D). Although a threshold of 126 mg/dL was set for carbohydrate feeding, however, that study did not determine if the sensor glucose was an accurate reflection of SMBG during exercise. More recently, CGM devices have been heavily scrutinized in the context of exercise due to the apparent lag between interstitial and blood glucose when the blood glucose concentration is changing rapidly.^9–12^ This lag is often attributed to the following: (1) physiologic lag affected by blood flow^10^; (2) sensor reaction time to glucose^13^; and (3) signal processing.^14^ There may also be a bias between the CGM and reference measurements due to the baseline signal (at a zero glucose concentration) generated by the CGM sensors. These discrepancies between CGM devices and SMBG may impact insulin-dosing decisions and carbohydrate replacement, particularly around exercise and during meals when glucose levels can change rapidly. In line with this, Biagi et al.^15^ reported an increase in MARD from 9.5% to 16.5% during aerobic exercise, while Moser et al.^16^ demonstrated a further increase in MARD (∼20%) when blood glucose levels dropped into the hypoglycemic range with exercise. Interestingly, Adolfsson et al.^17^ reported that MARD values also tended to increase with higher intensity (e.g., football, cross-country skiing) compared with lower intensity (e.g., golf) activities in adolescents with T1D. However, this has not been consistently observed in studies examining intermittent high-intensity exercise,^18,19^ likely because glucose levels tend not to change as dramatically because of a rise in glucose counterregulatory hormones and lactate levels.^20^

The primary purpose of this study was to assess the accuracy of newer CGM technology compared with SMBG during prolonged aerobic exercise and the meal postexercise in adults living with T1D. Our hypothesis is that CGM accuracy and MARD values will deteriorate during prolonged, steady-state moderate-intensity exercise and during the meal challenge postexercise.

## Methods

### Study participants

The present study conformed to the standards set by the Declaration of Helsinki and was approved by the Research Ethics Board at York University. The study was registered at clinicaltrials.gov in 2017 (identifier: NCT03130101). A total of 17 individuals (4 males, 13 females) with T1D were recruited for the study. The inclusion criteria included 17–65 years of age; duration of diabetes >1 year; using insulin pump therapy (OmniPod^®^ Insulin Management System; Insulet, Billerica, MA) for at least 1 month; a total daily insulin dose of at least 0.25 U/kg; and glycated hemoglobin (HbA_1c_) ≤9.9% (85 mmol/mol). The exclusion criteria included unpredictable hypoglycemia; not able to perform regular physical activity due to an injury; having conditions that may make exercise unsafe (i.e., high blood pressure and late pregnancy); and/or physician diagnosis of active diabetic retinopathy or neuropathy. Written informed consent was obtained from all participants before study initiation.

### Experimental design

These are ancillary data from a previously published study,^21^ using CGM for a secondary analysis. All exercise testing took place at York University in the Clinical Human Exercise Laboratory. Participants took part in one preliminary visit and three experimental visits, in which 60 min of aerobic exercise was performed with different insulin basal rate settings to help mitigate the risk for hypoglycemia. The preliminary testing consisted of questionnaires, anthropometric measurements, and a test of peak oxygen consumption (VO_2_peak). The experimental visits included three moderate-intensity aerobic exercise bouts with different adjustments in basal insulin, completed in a randomized counterbalanced manner. Participants were asked to refrain from all forms of vigorous-intensity exercise (i.e., activities more than six metabolic equivalents) for 24 h before each visit and were asked to avoid alcohol and caffeine consumption during the monitoring period.

### Preliminary testing (visit 1)

During the preliminary testing, height, body mass, blood pressure, body fat percentage, and HbA_1c_ (A1cNow+; Roxon Medi-Tech Ltd., Québec, Canada) were measured. The VO_2_peak test was an incremental-to-maximum treadmill protocol and was measured using a portable metabolic system (K5; COSMED, Rome, Italy) and heart rate monitor (Polar Electro, Kempele, Finland). The metabolic unit measures breath-by-breath expired oxygen and carbon dioxide concentrations using oxygen sampling line, turbine flowmeter, harness, face mask, and head strap.

### Continuous glucose monitor

All participants were instrumented with a CGM sensor and transmitter (Dexcom G4^®^ with 505 algorithm or G5^®^, San Diego, CA) at least 24 h before testing and were provided with a receiver or mobile app to track glucose levels throughout the study. A total of 7 participants were trained on the operation of CGM and the remaining 10 participants were asked to continue using their own CGM throughout the study duration. Of the 17 participants, four used CGM with Dexcom G4 Platinum with 505 algorithm (started before the study) and the remaining (*n* = 13) used CGM with Dexcom G5 throughout the study. Participants wore CGM for 1 week at a time and were instructed to use their Personal Diabetes Manager (PDM) for SMBG using the built-in glucose meter and associated blood glucose test strips (FreeStyle Lite; Abbott Laboratories, Abbott Park, IL). Following an initial warm-up period of 2 h after sensor insertion, participants were advised to calibrate at least once every 12 h using their own PDM glucose meter. If experimental visits were scheduled more than 1 week apart, participants were asked to insert a new sensor at least 24 h before the next visit. All CGM data were uploaded to the “Healthcare Professional” Dexcom Clarity^®^ account and the data were later retrieved for analysis.

### Experimental sessions (visits 2–4)

All participants completed three prolonged aerobic exercise visits that were separated by at least 24 h and all sessions were pooled for this analysis. Participants were asked to consume the same lunch of their choice and take their usual mealtime bolus insulin (plus correction, if necessary), no later than 11:30AM. The three insulin adjustment strategies included (1) pump suspension (100%) at exercise start, for the duration of the activity; (2) a 50% basal rate reduction (BRR), set 90 min pre-exercise for the duration of the activity; and (3) an 80% BRR, set 90 min pre-exercise for the duration of the activity. At 1:30PM participants were reminded to reduce basal insulin until the end of exercise. Exercise began at 3:00PM and consisted of 60 min of moderate-intensity (45%–55% of VO_2_peak) walking/light jogging on a treadmill. The activity was broken down into four 15-min bouts with 5-min rest periods in between.

Capillary glucose was determined at 30 and 10 min pre-exercise, just before exercise onset, and every 15 min during exercise using the OmniPod PDM (Insulet) with built-in glucose meter (FreeStyle; Abbott Laboratories). Measurements were completed in duplicate and if the duplicate value differed from the first value by >10 mg/dL, a third SMBG was determined. For analysis purposes, if duplicates were performed, then the average of both SMBG values were used, whereas if triplicates were performed, the average of the closest two values was used. If hypoglycemia (<70 mg/dL) developed during exercise, participants were provided with 16 g of dextrose tablets (Dex4^®^; AMG Medical, Inc., Québec, Canada). Additional dextrose was provided if the initial 16 g did not restore blood glucose concentrations. Exercise was resumed when SMBG values reached >81 mg/dL.

In all conditions, basal insulin was resumed to the usual (100%) rate immediately following exercise end. Following a 30-min rest period post-exercise, all participants consumed a standardized meal containing ∼30–50 g of carbohydrates, ∼10–20 g of protein, and ∼5–15 g of fat (Lean Cuisine, Nestlé, CA), and SMBG levels were monitored for 90 min postmeal.

### Identification of time lag and sensor bias

**FIG. 1.**
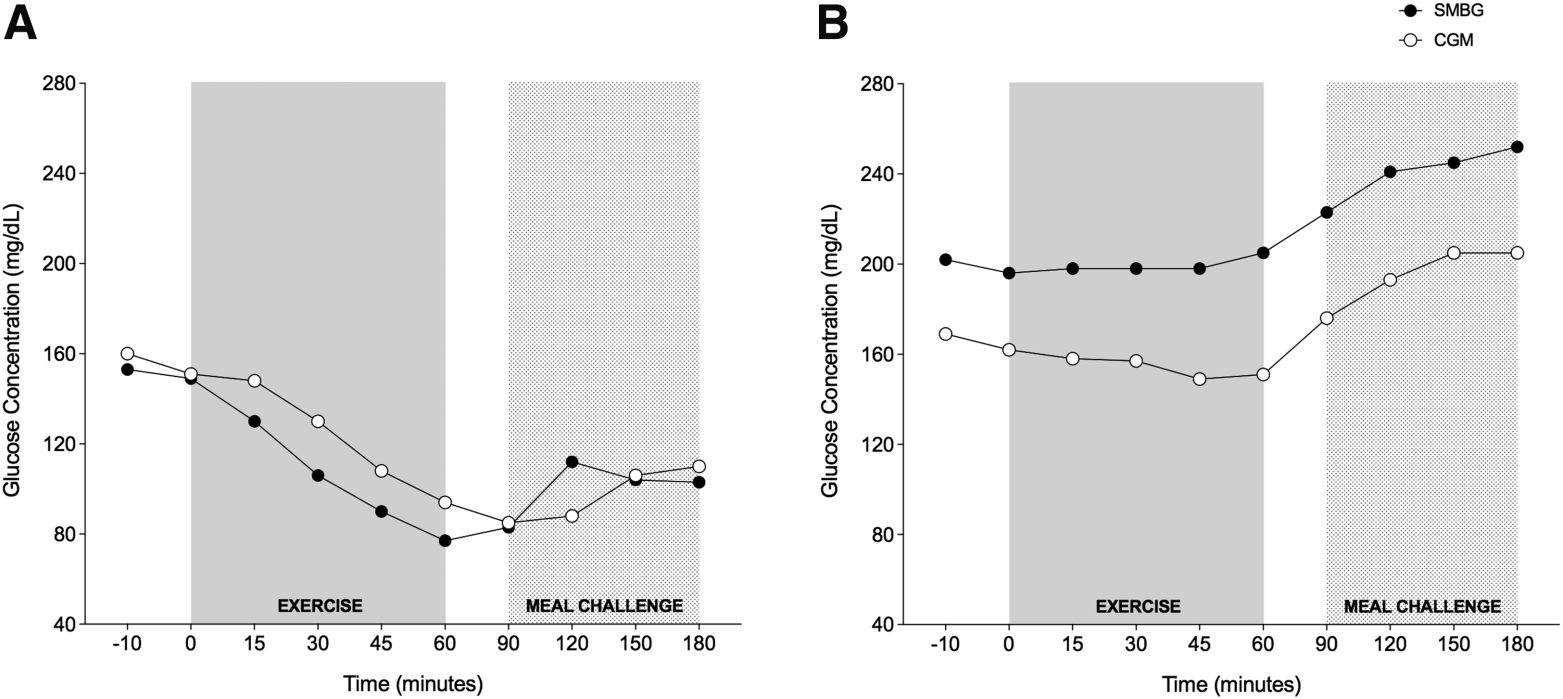
**(A)** Individual example of lag time (minutes) during the 80% BRR condition. **(B)** Individual example of bias (mg/dL/min) during pump suspension. Filled (black) circles represent SMBG measurements and white circles represent CGM measurements. BRR, basal rate reduction; CGM, continuous glucose monitoring; SMBG, self-monitoring of blood glucose.

The CGM uses mathematical models to convert raw sensor measurements to blood glucose concentrations. The measurements are based on interstitial glucose levels and the models correct for various error sources between the interstitial and blood glucose concentrations, including a physiological time lag and a bias (Fig. 1A, B).

A single compartment model is used:

\begin{align*}
{ \frac { dCGM }  { dt } } = \frac { { SMBG } }  { \tau } - \frac { { CGM } }  { \tau } + \varepsilon ,
\end{align*}

where *$\tau$* and *$\varepsilon$* represent the time lag and bias parameters and are identified through least squares fit. The numerical values of the derivative (or ROC) term *${ \frac { dCGM }  { dt } }$* are obtained with the Savitzky–Golay filter.^22^ The filter order and window size are set to 1 and 7, respectively. Least squares fit is used to find the unknowns *$\tau$* and *$\varepsilon$*. Three different sets of the unknowns were identified. The first set assumes that the lag and bias between the CGM and SMBG measurements are global over time. The latter two sets assume that the lag and bias are different during exercise and meal.

The pairing determination is based on the SMBG values that are paired with the closest CGM value within the next 5 min. Overall, there were 10 matched pairs of SMBG and CGM values for each participant across all three conditions. The “rest” time points include all the 10-min pre-exercise, exercise start, and premeal data. The “exercise” time points include 15, 30, 45, and 60 min of exercise. The “meal” time points include 30, 60, and 90 min following meal ingestion.

### Statistics

Statistical significance was set at *P* < 0.05 and the data are represented as mean ± standard error of mean as well as median and interquartile range. Absolute glucose concentrations during exercise and meal recovery were pooled across the three experimental conditions. A two-way repeated-measures analysis of variance was conducted to compare SMBG and CGM values during exercise and in the meal recovery postexercise. During rest, exercise, and recovery, there was no difference in Dexcom G4 and G5 glucose data when analyzed separately (data not shown), and therefore, all CGM data were pooled for all analyses. All of the graphics and statistical analyses were completed using GraphPad Prism Version 7.0 (GraphPad Software, CA). The MARD values were calculated using the absolute relative difference between the glucose meter value and CGM value divided by the glucose meter value, then multiplied by 100.

## Results

The anthropometric measurements for all 17 participants (4 males, 13 females) are summarized below. Participants recruited for this study were all adults (age 31 ± 10 years; mean ± standard deviation), height 168 ± 10 cm, weight 72 ± 10 kg, on insulin pump therapy, and in good glycemic control (HbA_1c_ 6.5% ± 0.5% or 47 ± 5 mmol/mol). The duration of diabetes was 14 ± 10 years and VO_2_peak was 41.6 ± 5.9 mL/(kg·min), indicative of good cardiometabolic fitness, but not of the elite level.

Since blood glucose measurements were completed in duplicate or triplicate for SMBG, the coefficient of variance (CV) was calculated during rest, exercise, and meal recovery. The CV between the two closest blood glucose readings was 2.2% ± 1.9% during rest, 2.3% ± 2.0% during exercise, and 2.2% ± 1.7% during the meal recovery.

Figure 1A represents the physiological time lag of one participant during the 80% BRR condition (overall lag of 19 min) and Figure 1B represents the CGM bias of one participant during the pump suspension condition (overall bias of 41 mg/dL/min).

**FIG. 2.**
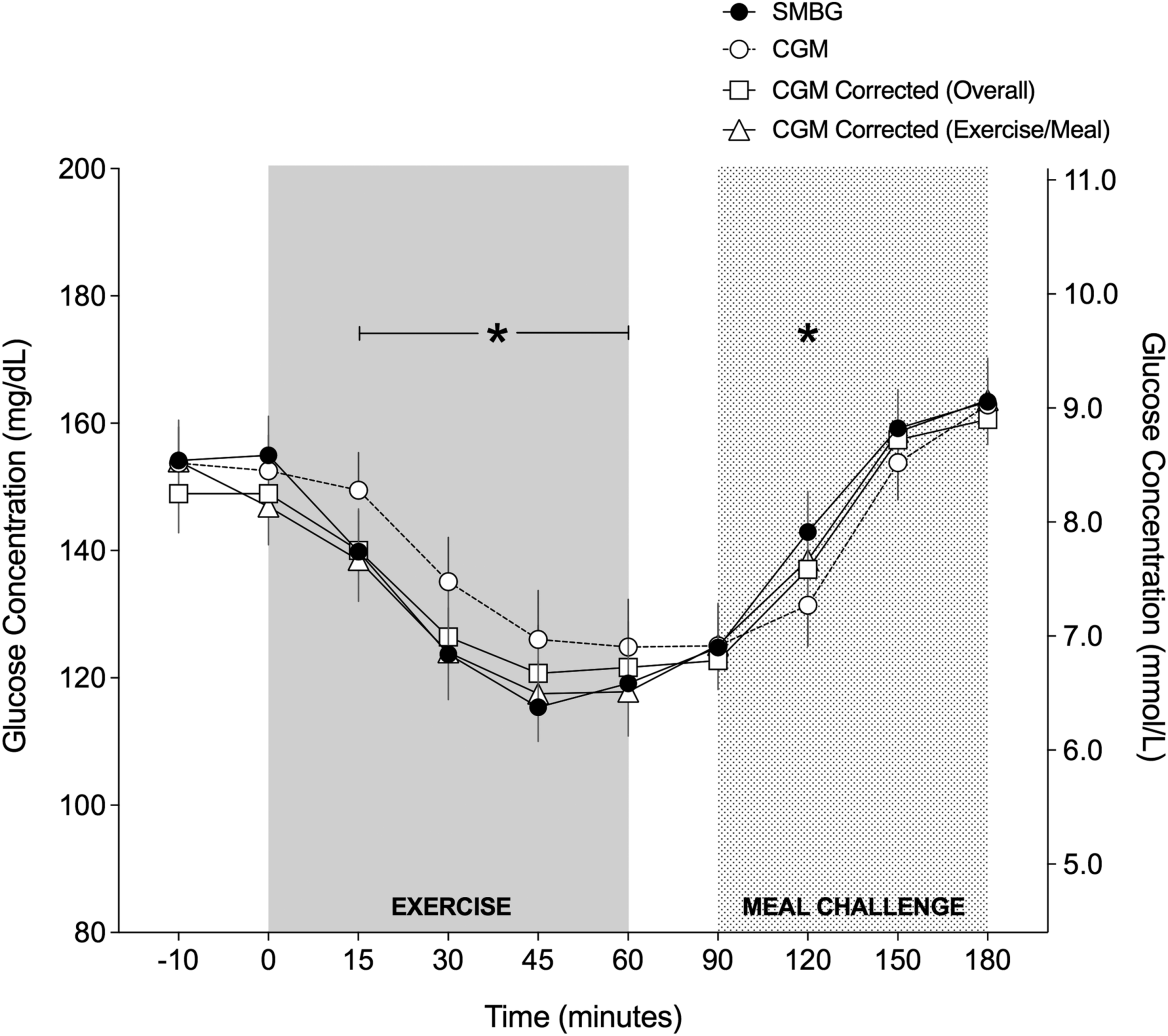
Absolute glucose values comparing SMBG, CGM, CGM corrected (overall), and CGM corrected during exercise and meal challenge. *Represents CGM different than SMBG (*P* < 0.05).

Figure 2 represents the mean glucose concentrations as measured by SMBG and CGM (including CGM corrected for lag and bias identified using the single-compartment model) during prolonged aerobic exercise and in the meal postexercise. From 15 min into exercise until the end of exercise, CGM values were significantly higher than SMBG values (*P* < 0.05). Overall, there were 12 documented episodes of hypoglycemia during the 51 total exercise sessions (i.e., 24%), as measured by SMBG, with a mean SMBG value of 60 ± 5.4 mg/dL. The corresponding CGM value during these documented hypoglycemic events was higher in all occasions, averaging 81 ± 10.2 mg/dL. In recovery, when blood glucose was rising, the average CGM value was significantly lower than the SMBG value 30 min postmeal (*P* = 0.001).

**FIG. 3.**
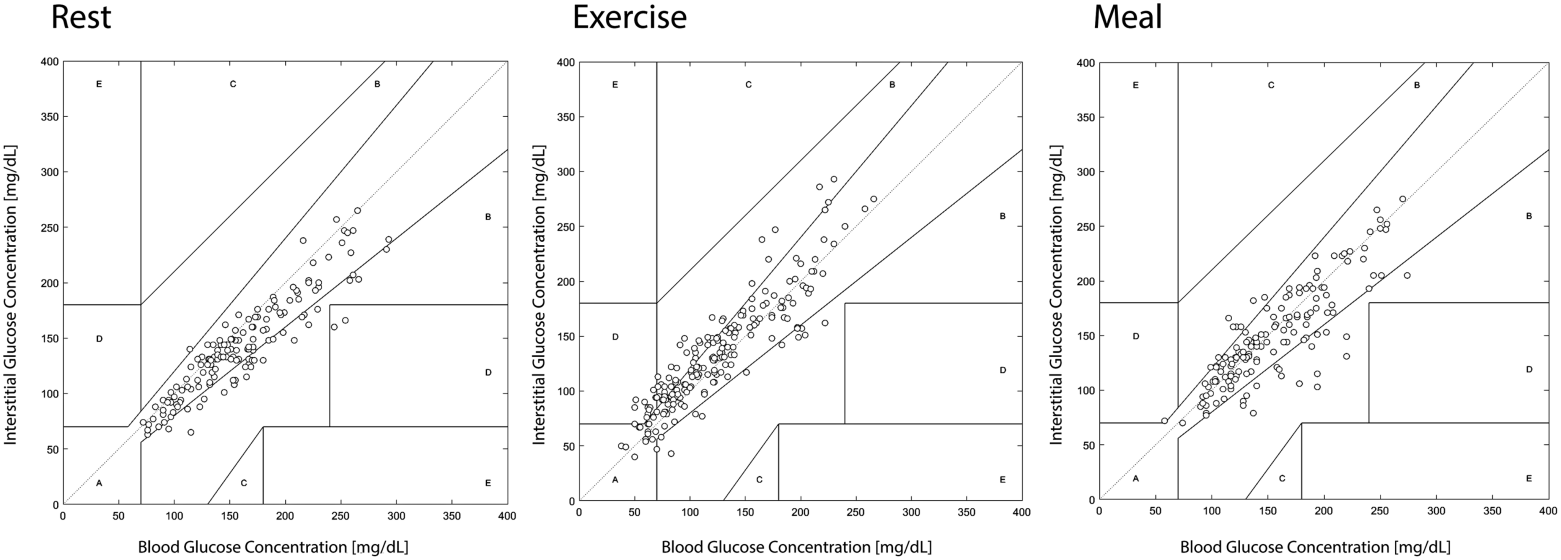
Clarke error grid comparing SMBG with CGM values during rest, exercise, and meal recovery.

Figure 3 represents Clarke error grid analyses for SMBG versus CGM at rest, exercise, and during the meal challenge. Based on regression analyses, the *r*-squared values for SMBG versus CGM were similar at *r*^2^ = 0.82, *r*^2^ = 0.82, and *r*^2^ = 0.73 during rest, exercise, and meal challenge, respectively (*P* > 0.05).

Table 1 represents the Clarke error grid analysis zones A–E that are used to depict the likelihood of inappropriate treatment-based CGM values compared with SMBG. For SMBG, compared with CGM, 99% of values fell in zones A and B during rest and the meal challenge, while 94% of values were in zones A and B during exercise.

**Table 1.** Clarke Error Grid Analysis

*Zones*	*SMBG vs. CGM*		
	*Rest*	*Exercise*	*Meal*
A	129/153 (84%)	141/204 (69%)	127/153 (83%)
B	23/153 (15%)	52/204 (25%)	25/153 (16%)
C	0/153 (0%)	0/204 (0%)	0/153 (0%)
D	1/153 (1%)	11/204 (5%)	1/153 (1%)
E	0/153 (0%)	0/204 (0%)	0/153 (1%)

Clarke error grid analysis zones A–E comparing SMBG versus CGM during rest, exercise, and meal postexercise. Zone A = values within 20% of glucose meter, zone B = points are outside of 20%, but would not lead to inappropriate treatment, zone C = points leading to unnecessary treatment, zone D = points indicate potentially dangerous failure to detect hypo- or hyperglycemia, and zone E = points that would confuse treatment of hypo- for hyperglycemia and vice versa.

CGM, continuous glucose monitoring; SMBG, self-monitoring of blood glucose.

Table 2 represents the three sets of lag and bias during exercise, meal, and overall for all three conditions as well as pooled. During exercise, the pooled lag time (τ) across all three conditions was 12 ± 11 min, while during mealtime, the pooled lag time was estimated to be 11 ± 13 min. The overall lag time with exercise and meal combined was 10 ± 8 min. During exercise, the pooled bias (ɛ) across all conditions was −7 ± 19 mg/dL/min and during the meal, the pooled bias was −0.02 ± 21 mg/dL/min. The overall bias with exercise and meal combined was −2 ± 18 mg/dL/min.

**Table 2.** Lag and Bias Across All Conditions Separately and Pooled

*Condition*	*Exercise only*		*Meal only*		*Overall*	
	*Lag*	*Bias*	*Lag*	*Bias*	*Lag*	*Bias*
80% BRR	11.7 ± 13.2 (3.2)	−5.4 ± 14.3 (3.5)	12.5 ± 18.3 (4.4)	−3.9 ± 19.7 (4.8)	9.6 ± 8.1 (2.0)	−2.2 ± 15.2 (3.7)
50% BRR	12.6 ± 12.5 (3.0)	−10.5 ± 20.9 (5.1)	8.9 ± 8.6 (2.1)	5.5 ± 22.0 (5.3)	10.5 ± 9.6 (2.3)	−4.1 ± 16.5 (4.0)
Pump suspension	10.1 ± 8.6 (2.2)	−3.6 ± 21.8 (5.5)	10.2 ± 9.0 (2.2)	−1.8 ± 21.1 (5.3)	9.3 ± 7.6 (1.9)	−0.8 ± 21.7 (5.4)
Pooled	11.5 ± 11.4 (1.6)	−6.6 ± 19.1 (2.7)	10.6 ± 12.7 (1.8)	−0.02 ± 21.0 (3.0)	9.8 ± 8.3 (1.2)	−2.4 ± 17.6 (2.5)
Pooled 95% CI	8.2–14.8	−12.0 to −1.2	7.0 to 14.1	−6.0 to 5.9	7.5 to 12.2	−7.4 to 2.6

Lag (τ in minutes) and Bias (ɛ, in mg/dL/min) separated during exercise and meal for all conditions as well as pooled data.

*Note:* Data represent mean ± standard deviation (standard error of mean).

BRR, basal rate reduction; CI, confidence interval.

Table 3 represents MARD during exercise and during the meal for all three conditions, as well as all conditions pooled. During exercise, the MARD percent across all three conditions was 13 (6–22)% and during the meal, the pooled MARD was 8 (5–14)%. The overall MARD during rest, exercise, and meal combined was 11 (7–18)%.

**Table 3.** Mean Absolute Relative Difference Percentage

*Condition*	*Exercise-only MARD*	*Meal-only MARD*	*Overall MARD*
80% BRR	6.7 (5.5–18.5)	7.6 (4.9–13.8)	9.2 (5.9–15)
50% BRR	11.1 (6.8–21.1)	6.6 (3.6–11.8)	9.3 (7.2–15.4)
Pump suspension	20.1 (17.9–30.5)	9.7 (5.6–16.7)	15.6 (10.6–21.6)
Pooled	12.9 (6.3–22.2)	7.6 (4.5–13.5)	11.0 (7.4–17.5)

MARD during exercise only and meal only for all three conditions, as well as pooled data.

*Note:* Data represent median (interquartile range).

MARD, mean absolute relative difference.

Table 4 shows the concurrence between CGM readings and SMBG for all three conditions. Specifically, at the lower end, the majority of more severe hypoglycemia (i.e., <60 mg/dL) reported by the CGM during the exercise sessions was found to be a false positive (i.e., false alarm) or in fact only mild (60–80 mg/dL) hypoglycemia. At the opposite end, glucose values in the hyperglycemic range (>200 mg/dL) were underestimated during the exercise sessions.

**Table 4.** Concurrence Between Continuous Glucose Monitoring Readings and Self-Monitoring of Blood Glucose for All Three Conditions

*No. of paired CGM-SMBG*			*CGM [mg/dL]*	*SMBG [mg/dL]*																							
				*<40*			*40–60*			*60–80*			*80–120*			*120–160*			*160–200*			*200–250*			*250–300*		
*R*	*E*	*M*		*R*	*E*	*M*	*R*	*E*	*M*	*R*	*E*	*M*	*R*	*E*	*M*	*R*	*E*	*M*	*R*	*E*	*M*	*R*	*E*	*M*	*R*	*E*	*M*
0	1	0	<40	–	–	–	–	100	–	–	–	–	–	–	–	–	–	–	–	–	–	–	–	–	–	–	–
0	7	0	40–60	–	14.3	–	–	–	–	–	71.4	–	–	14.3	–	–	–	–	–	–	–	–	–	–	–	–	–
9	17	5	60–80	–	–	–	22.2	17.6	20.0	44.4	64.7	20.0	33.3	17.6	40.0	–	–	20.0	–	–	–	–	–	–	–	–	–
33	64	37	80–120	–	–	–	–	4.7	–	18.2	32.8	–	63.6	50.0	62.0	–	12.5	24.4	–	–	13.5	–	–	–	–	–	–
61	56	55	120–160	–	–	–	–	–	–	–	–	–	21.3	33.9	18.2	59.0	53.6	60.0	20.0	8.9	16.4	–	3.6	5.4	–	–	–
31	37	32	160–200	–	–	–	–	–	–	–	–	–	–	–	3.1	19.4	40.5	15.6	58.1	45.9	68.8	22.6	13.5	13.5	–	–	–
14	11	17	200–250	–	–	–	–	–	–	–	–	–	–	–	–	–	–	–	14.3	45.5	17.6	78.6	54.5	58.8	7.1	–	23.5
2	7	4	250–300	–	–	–	–	–	–	–	–	–	–	–	–	–	–	–	–	–	–	50.0	71.4	25.0	50.0	28.6	75.0

The table is arranged by each SMBG range and tabulates for each range of SMBG readings, the percentage of paired CGM values that were in the identical glucose range (shaded diagonal), as well as those reference values that were in glucose ranges above and below the paired SMBG readings.

E, exercise; M, meal; R, rest.

Table 5 shows the concurrence between ROC of CGM and SMBG readings for all three conditions. The majority of ROC values were found to be within [−2, +2] mg/(dL·min) range. Overall, a significant percentage of the ROC values were above or below the estimated values.

**Table 5.** Concurrence Between Continuous Glucose Monitoring Rate of Change (ROC) and Self-Monitoring of Blood Glucose ROC for Three Different Conditions

*No. of paired CGM-SMBG*			*CGM ROC [mg/dL/min]*	*SMBG ROC [mg/(dL·min)]*																	
				*−3, −2*			*−2, −1*			*−1, 0*			*0, 1*			*1, 2*			*2, 3*		
*R*	*E*	*M*		*R*	*E*	*M*	*R*	*E*	*M*	*R*	*E*	*M*	*R*	*E*	*M*	*R*	*E*	*M*	*R*	*E*	*M*
0	5	0	*−*3, *−*2	–	60	–	–	20	–	–	20	–	–	–	–	–	–	–	–	–	–
9	31	4	*−*2, *−*1	33	7	–	56	55	–	–	36	75	11	3	25	–	–	–	–	–	–
71	107	47	*−*1, 0	1	–	–	21	9	–	56	67	49	22	22	47	–	2	4	–	–	–
66	53	68	0, 1	–	–	–	6	4	–	24	19	9	59	72	78	11	6	13	–	–	–
4	3	27	1, 2	–	–	–	–	–	–	–	–	–	75	67	70	25	–	30	–	33	–
0	1	4	2, 3	–	–	–	–	–	–	–	–	–	–	–	50	–	100	50	–	–	–

The table is arranged by each SMBG ROC range and tabulates for each SMBG ROC range, the percentage of paired CGM ROC in the identical range (shaded diagonal), as well as those reference values that were in ranges above and below the paired SMBG readings.

ROC, rate of change.

## Discussion

In the past, CGM devices were not considered for nonadjunctive use, primarily due to the concern of inaccuracy that could lead to potentially inappropriate treatment and insulin-dosing decisions.^23^ However, as CGM technology and accuracy improve over time, several devices are being recognized as the standard of care for managing T1D and gaining nonadjunctive status, so that insulin-dosing decisions and hypoglycemia management can be made without confirmatory SMBG.^3,5^ Since most studies are carried out under tightly controlled conditions in laboratory settings and few studies have assessed the accuracy of CGM and flash glucose monitor devices in outpatient settings,^24,25^ future studies should focus on comparing the newest and most advanced glucose-monitoring technology in outpatient settings with a greater emphasis on exercises of varying intensities and durations.

Previous literature has looked at CGM performance during both aerobic and anaerobic exercise^12,15,18,19^; however, to our knowledge, this is the first study to measure CGM accuracy during a structured 60-min aerobic exercise bout using primarily newer Dexcom G5 technology (*n* = 13). The remaining participants (*n* = 4) wore Dexcom G4 Platinum with updated software and 505 algorithm. In addition, our analysis showed no difference with the G4^®^ data removed; therefore, all CGM data were pooled. In the present study, MARD values were higher during exercise [13 (6–22)%] compared with the meal recovery [8 (5–14)%]. More specifically, when we separated the data by BRR condition (Table 3), we found the highest MARD in the pump suspension condition [20 (18–31)%], compared with the 80% and 50% BRR conditions [7 (6–19)% and 11 (7–21)%, respectively]. As expected, the greatest drop in blood glucose concentration coincided with the highest MARD, which was apparent in the pump suspension condition. These findings are also in agreement with the findings of Moser et al. that found increased MARD and a physiological lag time of ∼3–12 min during exercise.^12^ As such, our findings coincide with previously published literature suggesting sensor accuracy may still be compromised, particularly during prolonged exercise when glucose levels tend to change rapidly.^12,15,19,26^ During exercise, particularly at both glucose extremes (<60 and >200 mg/dL), we found a greater discrepancy in CGM values relative to SMBG values. Therefore, to ensure safety, our recommendation for individuals with T1D is to use CGM in addition to a standard blood glucose meter during physical activity.

The present study assessed the accuracy of CGM compared with SMBG during exercise and in meal recovery. First, we found that CGM had good congruence with SMBG during rest, likely because the CGM was calibrated to this glucose meter. Second, we found that mean CGM values underestimated the drop in mean glucose during exercise and lagged significantly behind SMBG. It is important to highlight that in those individuals who developed hypoglycemia during exercise, as measured by SMBG, the CGM overestimated glucose by about 20 mg/dL, on average, with very few of the CGM readings in the hypoglycemic range (i.e., 80% of the time, the CGM was measuring in the euglycemic range). Therefore, based on our findings, we suggest setting a higher CGM threshold to initiate carbohydrate feeding to treat (i.e., 90 mg/dL) or prevent (i.e., 120 mg/dL) hypoglycemia, if the CGM displays downward trend arrows.

It is worth noting that we found significant variation within the identified lag and bias values during exercise and these variations may be due to intersubject or sensor variability.^10^ These findings may have critical implications for the development of hybrid closed-loop or automated insulin delivery systems for exercise since these devices may inadvertently overdeliver insulin when plasma glucose is dropping, but sensor glucose fails to drop or may even rise.^26^ In contrast, for the meal response after exercise, the delayed rise in sensor glucose may result in insulin underdelivery compared with plasma glucose and/or SMBG values. To overcome some of these challenges, Turksoy et al.^27^ determined that incorporating wearable technology with automated insulin delivery can better detect the changes in glucose levels and is showing promise in the prevention of hypoglycemia during exercise.

A number of studies have looked at CGM accuracy during exercise; some using SMBG as a reference,^17^ while others compare plasma glucose as the reference, using a glucose analyzer.^18,19,28^ The laboratory standard for CGM accuracy is most commonly a glucose analyzer (YSI; Xylem, Inc., OH)^29,30^; however, we chose SMBG as the reference in this study. Interestingly, according to a recent study that used capillary blood glucose concentration as a reference for CGM accuracy compared with venous blood found that SMBG as the reference was associated with significantly lower MARD values compared with plasma glucose as measured by a glucose analyzer (HemoCue measurement system, Ängelholm, Sweden).^31^ In a real-world setting, patients will not have glucose analyzers available to use as a reference during exercise, and therefore, SMBG is the most common reference measurement.

Measuring CGM accuracy may be negatively impacted if the reference method is different from the method that is used to calibrate the CGM device.^31^ In the present study, all CGM calibrations were made using the participant's own PDM glucose meter. As such, SMBG and CGM values were not different during rest (*P* > 0.05), but CGM lag time was apparent, specifically during exercise. As can be observed in Figure 2, CGM lags behind SMBG during exercise and tends to underestimate the drop in glucose compared with SMBG. Unfortunately, we did not measure plasma glucose levels using a standardized glucose analyzer, so it is currently unclear whether the glucose meter was more accurate or if the drop in glucose as measured by CGM was a true underestimation of circulating plasma glucose concentrations. However, with the data that we collected, we found a low CV (%) between the two closest glucose readings for SMBG (2.2% ± 1.9%).

During exercise, as shown in Figure 3, 94% of the values are in zones A and B of the Clarke error grid. It is important to recognize that the regression analysis alone does not differentiate which points are in zones A to E. For example, the values that are in zone D (5% during exercise) could lead to failure to detect hypoglycemia or hyperglycemia. Despite a clear lag in glucose as measured by CGM during exercise, we found that sensor glucose levels had good congruency during the meal challenge.

This study has a number of limitations that should be acknowledged. First, we included only SMBG measurements and were lacking measurements from a glucose analyzer (i.e., YSI). Second, due to the frequent fingerstick glucose sampling during exercise, SMBG values were determined every 30 min during the meal challenge rather than every 15 min such as during exercise. Third, these ancillary data included only one form of exercise (i.e., aerobic) and future studies would benefit from assessing CGM lag time during various intensities and durations of activity. Finally, our study included the use of both Dexcom G4 with 505 algorithm and G5 technology, and therefore, future studies should conduct similar analyses in a real-world setting and use the latest CGM technology available.

In summary, CGM technology helps patients closely monitor glucose levels and make appropriate changes to prevent dysglycemia. Numerous studies have reported that regular use of CGM lowers the time spent in hypoglycemia, and improves HbA_1c_ and quality of life in both children and adults with T1D.^32–35^ However, we conclude that the accuracy of newer CGM technology remains negatively impacted during prolonged aerobic exercise and patients need to be aware of this potential CGM time delay. More specifically in our study, the CGM (using Dexcom G4 Platinum with 505 algorithm and G5 technology) lag time was 12 ± 11 min behind SMBG readings during exercise with a bias of −7 ± 19 mg/dL/min. Due to this clinically important delay in CGM versus SMBG, we suggest patients increase vigilance and perform more frequent fingerstick capillary glucose monitoring around exercise.
